# Personalized ß-lactam dosing in patients with coronavirus disease 2019 (COVID-19) and pneumonia: A retrospective analysis on pharmacokinetics and pharmacokinetic target attainment

**DOI:** 10.1097/MD.0000000000026253

**Published:** 2021-06-04

**Authors:** Ute Chiriac, Otto R. Frey, Anka C. Roehr, Andreas Koeberer, Patrick Gronau, Thomas Fuchs, Jason A. Roberts, Alexander Brinkmann

**Affiliations:** a Department of Pharmacy, University Hospital of Heidelberg; b Department of Pharmacy, Heidenheim General Hospital; c Department of Anesthesiology and Intensive Care Medicine, Heidenheim General Hospital; d Department of Anesthesiology and Intensive Care Medicine, Heidenheim General, Heidenheim, Germany; e University of Queensland Centre for Clinical Research, Faculty of Medicine, The University of Queensland; f Departments of Pharmacy and Intensive Care Medicine, Royal Brisbane and Women's Hospital, Brisbane, Australia; g Division of Anaesthesiology Critical Care Emergency and Pain Medicine, Nîmes University Hospital, University of Montpellier, Nîmes France.

**Keywords:** continuous infusion, coronavirus disease 2019, critical illness, PK/PD, ß-lactams, therapeutic drug monitoring

## Abstract

Pathophysiological changes are important risk factors for critically ill patients with pneumonia manifesting sub-therapeutic antibiotic exposures during empirical treatment. The effect of coronavirus disease 2019 (COVID-19) on antibiotic dosing requirements is uncertain. We aimed to determine the effect of COVID-19 on ß-lactam pharmacokinetics (PK) and PK target attainment in critically ill patients with a personalized dosing strategy.

Retrospective, single-center analysis of COVID-19 ± critically ill patients with pneumonia (community-acquired pneumonia or hospital-acquired pneumonia) who received continuous infusion of a ß-lactam antibiotic with dosing personalized through dosing software and therapeutic drug monitoring. A therapeutic exposure was defined as serum concentration between (c_ss_) 4 to 8 times the EUCAST non-species related breakpoint).

Data from 58 patients with pneumonia was analyzed. Nineteen patients were tested COVID-19-positive before the start of the antibiotic therapy for community-acquired pneumonia or hospital-acquired pneumonia. Therapeutic exposure was achieved in 71% of COVID-19 patients (68% considering all patients). All patients demonstrated c_ss_ above the non–species-related breakpoint. Twenty percent exceeded c_ss_ above the target range (24% of all patients). The median ß-lactam clearance was 49% compared to ß-lactam clearance in a standard patient without a significant difference regarding antibiotic, time of sampling or present COVID-19 infection. Median daily doses were 50% lower compared to standard bolus dosing.

COVID-19 did not significantly affect ß-lactam pharmacokinetics in critically ill patients. Personalized ß-lactam dosing strategies were safe in critically ill patients and lead to high PK target attainment with less resources.

**Key messages**

- There was no evidence of different ß-lactam pharmacokinetics, nor the need of higher doses in patients with COVID-19 and pneumonia.
- Recommended personalized dosing strategy of ß-lactams ensures sufficient serum drug concentrations.
- Less drug and saving of nursing time due to personalized dosing strategies might help to overcome drug shortages and increased workload.

## 1 Introduction

The pandemic of coronavirus disease 2019 (COVID-19) caused by the novel severe acute respiratory syndrome coronavirus 2 (SARS-CoV-2) presents an unprecedented challenge to identify effective treatment. Although a prominent part of lung injury may be caused by the virus, concerns over bacterial co-infection or superinfection require empirical coverage in patients with community-acquired pneumonia (CAP) or hospital-acquired pneumonia (HAP) without confirmed COVID-19.^[1,2]^ It is likely that the relevant bacterial pathogens in patients with COVID-19 and pneumonia are the same as in patients with pneumonia before the pandemic and therefore ß-lactams plus either a macrolide or a fluoroquinolone or as monotherapy are recommended.^[1,2]^

Effective antibiotic treatment is depended on sufficient concentrations at the site of infection.^[3–5]^ The local concentration in the lung may be particularly unpredictable in critically ill patients as compared to healthy humans.^[3,6]^ Pathophysiological changes in volume of distribution, drug clearance, and protein-binding can be significantly different in critically ill patients compared to what is observed in other patient groups.^[3,7–13]^ In COVID-19 patients with sepsis-related multiple organ dysfunction and/or a strong cytokine storm, pathophysiological changes remains poorly defined. It has been suggested, that organotropism might aggravate preexisting conditions.^[14]^ At the same time ß-lactams are prone to rapid changes in renal function and volume of distribution, due to their hydrophilic properties, short half-life, predominant renal clearance, low volume of distribution, and low intracellular penetration resulting in variable and unpredictable antibiotic serum concentration in critically ill patients with standard dosing.^[11]^ Therefore, antibiotic dosing in critically ill patients is highly challenging and, a more personalized approach to drug dosing, with consideration of pharmacokinetics in the individual patient and pathogen susceptibility, is required. Using dosing strategies, including application of dosing software and therapeutic drug monitoring (TDM), it is possible to ensure more patients achieve target drug exposures.^[15]^

From a pharmacokinetic (PK)/pharmacodynamic perspective, animal and preclinical studies have defined ß-lactams to be time-dependent. The time for which the free (unbound) antibiotic concentration is maintained above the minimum inhibitory concentration (MIC) is the determinant factor associated with bactericidal activity (*f*T > MIC).^[16]^ Hence, prolonged application such as continuous infusion (CI) represents a reasonable approach to maximize bacteriological and clinical response by maintaining concentrations throughout the dosing interval.^[16]^ TDM-guided prolonged application including CI of ß-lactams in critically ill patients which is advocated by national^[17–19]^ and international guidelines^[20]^ may improve outcome of critically ill patients.^[21–23]^ In addition, administration by CI has the potential to reduce expenses for labor and supplies.^[24–26]^

The primary objective of this study was to assess the implications of COVID-19 on the pharmacokinetics of ß-lactams in critically ill patients with pneumonia. The secondary objective was to determine whether a personalized dosing strategy, including application of dosing software, TDM, and CI, enables COVID-19 patients to achieve target ß-lactam exposures with less resources.

## 2 Methods

### 2.1 Study design and population

This was a retrospective, single-center analysis of critically ill patients with pneumonia who received TDM-guided CI of a ß-lactam antibiotic for CAP or HAP during the COVID-19 pandemic in Germany (02–05 2020). Pneumonia was defined by radiological, clinical and laboratory parameters (leucocytes, C-reactive protein and procalcitonin). The study was approved by the ethics commission of the University of Ulm, Germany (Project number 137/19 including amendment 06/2020).

### 2.2 Study procedures

Patients received TDM-guided intravenous CI of a ß-lactam antibiotic according to a standardized protocol that derived from years of clinical experience and the routine use of PK simulations.^[27]^ This approach consisted of a loading dose (15-minute infusion, 50% standard dose) followed by immediate CI.^[27]^ Empiric daily doses were calculated by a clinical dosing software (the CADDy program)^[28]^ and subsequently adjusted by TDM. A therapeutic exposure was defined as piperacillin concentration of 32 to 64 mg/L, ampicillin concentration of 16 to 32 mg/L, cefotaxime concentrations of 16 to 32 mg/L, cefepime concentrations of 16 to 32 mg/L and meropenem concentrations of 8 to 16 mg/L corresponding to 4 to 8 times the non–species-related breakpoints of the European Committee on Antimicrobial Susceptibility Testing's MIC90 data (http://www.eucast.org/clinical_breakpoints: ampicillin 4 mg/L, piperacillin 8 mg/L, cefotaxime 2 mg/L, cefepime 4 mg/L, meropenem 2 mg/L).^[29]^ TDM-guided dose adjustments and consecutive TDMs were advised and supervised by trained clinical pharmacists. Blood samples were collected using the indwelling arterial catheter with under steady-state conditions. Adjustment to microbiological data and resistance testing was performed as soon as relative findings were available.

Total concentrations were analyzed using a validated high-performance liquid chromatography (HPLC).^[30]^ TDM-data were available and reported 2 to 4 hours after the blood sample arrived in the laboratory.

### 2.3 Pharmacokinetic analysis

A 1-compartment model was used to perform PK analyses because ß-lactams have a small volume of distribution, low protein-binding, and are essentially excreted by the kidneys. Pharkin 4.0 was used to perform PK simulation (http://www.pharkin.de). Serum concentrations in steady-state (c_ss_) were the observed values. ß-lactam clearance was calculated using the following equation: $CL[L\cdot h^{-1}]=\frac{dose[mg]}{24h}\cdot C_{SS}^{-1}[mg\cdot L^{-1}]$. Creatinine clearance (CrCL) was calculated using the Cockcroft-Gault equation.^[31]^

### 2.4 Pharmacoeconomic analysis

Costs associated with drug acquisition, preparation, administration process (including loading dose, preparation time, administration time, cost of materials required for the drug preparation and administration), and serum drug concentration measurement using HPLC (including acquisition cost, cost of materials required for HPLC, labor costs) were compared with standard bolus dosing for the same time period. Median daily doses and median number of administrations used for calculations are the observed values. Nursing times spent on preparation and administration and their costs as well as cost of HPLC measurement and drug cost were extrapolated from a previous time-motion study conducted at our institution.^[32]^

### 2.5 Statistical analysis

PK-, TDM-, and patient data were processed anonymously and included into a Microsoft Excel database (Microsoft Corp., Version 16.16.18). All calculations and statistical analysis were performed using IBM SPSS Statistics version 26 software (IBM, Armonk, NY). Discrete variables are expressed as counts (percentage) and continuous variables as means ± standard deviation (SD) or median (IQR). Man–Whitney test, Wilcoxin-W, and Kruskal-Wallis test were performed to evaluate statistical significance. Significant levels were considered as *P* ≤ .05.

## 3 Results

During the COVID-19 pandemic in Germany, 509 cases were registered in the district of Heidenheim, Germany (130,000 inhabitants) of which 40 cases died from COVID-19. In that time, 58 patients with pneumonia (CAP 27 [33%], HAP 54 [67%]) were treated in our intensive care unit, of which 19 patients were tested positive for SARS-Cov19 during hospital stay and were compared to the other 39 patients.

**Figure 1:**
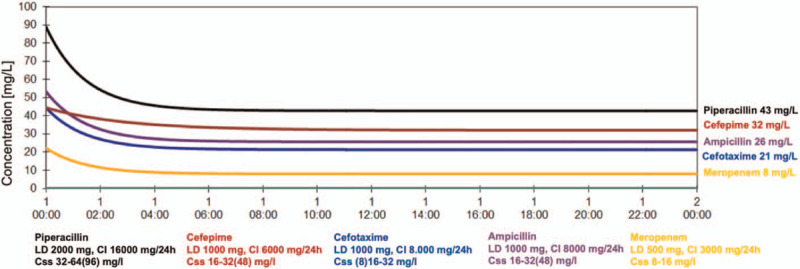
ß-lactam target concentration. Continuous infusion with a loading dose in a standard patient (50-year-old 75 kg male with CrCL of 100 mL/min).

In total, 220 samples were analyzed (101 COVID-19 samples, 119 non-COVID-19 samples) of whom 99 samples where obtained within the first 48 hours of therapy and 121 samples throughout the treatment course (day 1: 52, day 2: 47, day 3: 45, day 4: 30, day 5: 19, day 6: 15, day 7: 5, day 8: 4). The demographic and general clinical characteristics of the patients were well balanced between the groups (Table 1). Briefly, the study population was relatively old (mean age 71 years, SD 12) and had reduced renal function on the day of inclusion (median 53 CrCL mL/minute, range 11–158 mL/minute). The median (range) sequential organ failure assessment score was 6 (1–17). Of the 58 patients, 10 (17%) were treated with ampicillin, 28 (48%) with piperacillin, 9 (16%) with meropenem, 16 (28%) with cefotaxime, and 17 (29%) with cefepime. TDM was performed median 2 times per treatment (ampicillin 30, piperacillin 60, cefotaxime 38, cefepime 54, meropenem 38; given in number of serum concentrations). Target serum concentrations according to protocol simulated in a standard patient (50-year-old 75 kg male with CrCl of 100 mL/min) with loading dose and with standard dosing given as CI are shown in Figure 1.

**Table 1:** Patient characteristics^∗^.

Characteristics	All patients (n = 58)	COVID-19 (n = 19)	Non-COVID-19 (n = 39)
Age, y, mean (SD)	71 (12)	74 (10)	70 (12)
Weight, kg, mean (SD)	80 (18)	81 (19)	79 (18)
Height, cm, mean (SD)	171 (10)	169 (10)	173 (9)
Body mass index, kg/m^2^, mean (SD)	27 (6)	28 (6)	27 (6)
Body mass index, kg/m^2^ ≥ 30 (%)	14 (24%)	6 (32%)	8 (21%)
Sex, male/female (%)	34 (59%) / 23 (41%)	8 (42%)/ 11 (61%)	26 (67%) / 13 (33%)
CrCL on day of inclusion, mL/min, median (IQR)	53 (58)	51 (43)	56 (58)
CVVHD (%) on day of inclusion	11 (14%)	5 (19%)	6 (11%)
CRP in serum on day of inclusion, mg/dL, median (IQR)	202 (137)	216 (184)	201 (123)
Leukocytes, 10^9^ cells/L, on day of inclusion (IQR)	11.6 (9.0)	11.6 (6.7)	11.7 (9.2)
SAPS on day of inclusion (IQR)	42 (15)	42 (12)	42 (16)
SOFA Score on day of inclusion (IQR)	6 (6)	8 (8)	6 (5)
Procalcitonin (IQR)	1.24 (4.88)	0.88 (2.40)	1.49 (7.01)
ICU mortality (%)	15 (26%)	7 (37%)	8 (21%)
Mechanical ventilation on day of inclusion (%)	51 (63%)	22 (81%)	29 (54%)
Diagnosis, CAP/HAP, on day of inclusion (%)	27 (33%)/54 (67%)	8 (30%)/23 (85%)	19 (35%)/31 (57%)

### 3.1 Pharmacokinetic analysis

Therapeutic drug exposure was realized in 71% of COVID-19 patients (68% considering all patients). The minimum of the target range was realized in 92% of COVID-19 patients (91% considering all patients). All patients achieved c_ss_ above the non–species-related breakpoints. Twenty percent of the patients exceeded serum concentrations above the target range (24% considering all patients). The data describing the achievement of PK targets with TDM-guided dosing are shown in Table 2.

**Table 2:** Pharmacokinetic target attainment in critically ill patients with a personalized dosing strategy including dosing software, continuous infusion and therapeutic drug monitoring. Values are given in relative incidence for COVID-19 patients (all patients).

			Probability of target attainment				
	Non–species-related breakpoint	Target range of c_ss,_ mg/L	Target range of c_ss_	< Non–species-related breakpoint	>2 x Non–species-related breakpoint	> Minimum target c_ss_	> Maximum target c_ss_
Ampicillin	4	16–32	80% (63%)	0% (0%)	100% (100%)	90% (90%)	10% (27%)
Cefotaxime	4^∗^	16–32	69% (68%)	0% (0%)	100% (100%)	87% (84%)	19% (16%)
Piperacillin	8	32–64	55% (45%)	0% (0%)	100% (100%)	85% (92%)	40% (37%)
Cefepime	8	16–32	70% (61%)	0% (0%)	100% (100%)	96% (94%)	26% (33%)
Meropenem	2	8–16	92% (91%)	0% (0%)	100% (100%)	97% (97%)	6% (5%)

**Figure 2:**
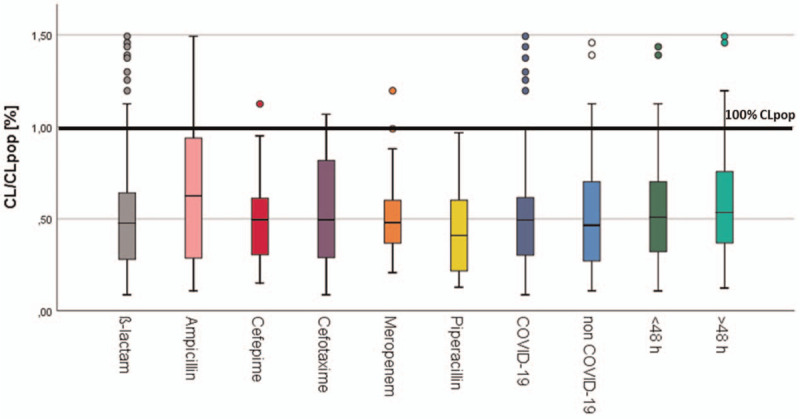
ß-lactam clearance. Distribution of ß-lactam clearance (CL) compared to ß-lactam clearance in a standard patient (CL_pop_) given in percentage of all patients (n = 58), patients with COVID-19 (n = 19), patients without COVID-19 (n = 39), patients with sampling in the first 48 h, and after 48 h (n = 40). Man–Whitney and Kruskal-Wallis test were performed to evaluate statistical significance. Significant levels were considered as *P* ≤ .05.

The median ß-lactam clearance was 50% compared to population clearance (Fig. 2). No significant difference regarding the ß-lactam, time of sampling, or present COVID-19 infection was observed (Fig. 2). No augmented clearance in the first 48 hours was observed (Fig. 2).

### 3.2 Economic impact of personalized dosing

On average, median daily dose in this study was 50% compared to standard bolus dosing (ampicillin 4000 mg, piperacillin 8000 mg, cefotaxime 4150 mg, cefepime 2100 mg, meropenem 2000 mg; given as median daily doses). In accordance with this median number of administrations in this study was half the administrations with standard bolus dosing (ampicillin 2.0, piperacillin 2.0, cefotaxime 2.1, cefepime 1.0, meropenem 2.0; given as median number of administrations) resulting in shorter nursing times (preparation time and administration time). On average, median nursing time in a seven-day treatment course was 50% less (ampicillin 72 minutes [125 minutes], piperacillin 72 minutes [167 minutes], cefotaxime 74 minutes [125 minutes], cefepime 41 minutes [125 minutes], meropenem 72 min [125 minutes]; given as median nursing time with personalized dosing [with standard bolus dosing]). Total costs per treatment course were 36% lower compared to standard bolus dosing (Table 3).

**Table 3:** Comparison of treatment course costs for a personalized dosing strategy including dosing software, CI, and TDM, in critically ill patients with pneumonia (7-day treatment course, 2 samples for concentration measurement) compared to standard bolus dosing.

	Ampicillin		Piperacillin		Cefotaxime		Cefepime		Meropenem	
	CI	Bolus	CI	Bolus	CI	Bolus	CI	Bolus	CI	Bolus
TDM	21.34 €	– €	21.34 €	– €	21.34 €	– €	21.34 €	– €	21.34 €	– €
Drug aquisition	127.48 €	178.47 €	336.09 €	627.37 €	267.52 €	362.45 €	266.12 €	670,61 €	532.47 €	745.45 €
Material cost	13.29 €	26.25 €	13.29 €	35.00 €	13.72 €	26.25 €	7,56 €	26.25 €	13.29 €	26.25 €
Preparation time	22.77 €	40.11 €	22.77 €	53.48 €	23.52 €	40.11 €	12,84 €	40.11 €	22.77 €	40.11 €
Administration time	3.93 €	6.09 €	3.93 €	8.12 €	4.06 €	6.09 €	2,20 €	6.09 €	3.93 €	6.09 €
Total	189 €	251 €	397 €	724 €	330 €	435 €	310 €	743 €	594 €	818 €

## 4 Discussion

To our knowledge, this is the first reported experience of CI of ß-lactams in critically ill patients with pneumonia (CAP/HAP) and COVID-19. We found that therapeutic target attainment is high with a personalized dosing strategy, demonstrating a median target attainment of 68% independent of actual COVID-19-status. Considering that positive effects of CI of β-lactams on patient outcome have been repeatedly reported,^[21,33–35]^ this study emphasizes the present recommendations on antibiotic dosing in critically ill patients and supports the use of CI with dosing personalized through dosing software and TDM to minimize the likelihood of clinical failure or adverse effects.

In accordance with previous data, we identified pathophysiological changes presented as reduced ß-lactam clearance compared to non-critically ill patients.^[3,12]^ Since previous findings indicate that SARS-COV-2 has an organotropism beyond respiratory tract including the kidneys which might aggravates preexisting conditions, patients with COVID-19 might have additional pathophysiological changes affecting ß-lactam concentrations.^[14]^ However, we found that no significant PK differences occurred between patients with COVID-19 and patients without. All patients achieved *f*c_ss_ above 2 times the non–species-related breakpoint with half the standard daily dose when administering by TDM-guided CI. In contrast to our findings, antibiotic standard dosing without TDM showed a remarkably high PK-target nonattainment.^[11]^ In the DALI study, only 35% and 60% of the patients achieved 100% *f*T > 4x MIC (ampicillin 22%, piperacillin 30%, cefepime 71%, meropenem 42%) and 100% *f*T > 1x MIC ampicillin 33%, piperacillin 67%, cefepime 79%, meropenem 70%), respectively.^[11]^ Whereas β-lactam *f*c_min_/MIC > 1.3 was found to be a significant predictor of a positive clinical outcome in critically ill patients with gram-negative blood stream infections.^[36]^

In patients with impaired renal function, the relationship between CrCL and decreased drug amount may not come as a surprise as previous studies have already demonstrated the correlation between CrCL and clearance of β-lactam antibiotics.^[12]^ However, previous findings sometimes postulate a normal ß-lactam clearance independent of the renal function in critically ill patients in the beginning of the infection resulting in target nonattainment within the first 48 hours.^[37]^ Our data do not support these considerations and suggest dose adjustment according to renal function from the beginning to avoid very high ß-lactam concentrations. Imani et al^[38]^ clearly demonstrated increased neuro- and nephrotoxicity with irrationally high PK targets (>6–8× MIC in a worst case scenario eg, pseudomonas). Impaired renal function may cause for instance high piperacillin serum concentrations (>96 mg/L) which may readily exacerbate a preexisting renal dysfunction resulting in acute kidney injury.^[12]^ The potentially nephrotoxic effects of piperacillin are of special concern when a combination therapy (ie, vancomycin) is pursued.^[39]^ Standard dosing in the critically ill patient may indeed induce both impaired renal function and a higher incidence of neurotoxicity.^[40]^ Neurotoxicity and nephrotoxicity were demonstrated in several studies for cefepime, piperacillin, and meropenem.^[38,41,42]^ With regard to adverse effects, a particular attention should be given to possible antibiotic toxicity in patients experiencing unexplained neurological manifestations or renal failure.^[38]^ In our study, 24% of the patients exceeded the therapeutic target range (ampicillin 27%, piperacillin 37%, cefotaxime 16%, cefepime 33%, meropenem 5%). c_ss_ > 96 mg/L: 5%, cefotaxime c_ss_ > 48 mg/L: 8%, cefepime c_ss_ > 48 mg/L: 4%, meropenem c_ss_ > 24 mg/L: 0%). Therefore, personalized dosing might help to avoid a potentially harmful effect of very high ß-lactam concentrations in patients.

From an economic point of view, less drug to maintain concentrations above the MIC by administering ß-lactams as CI can decrease drug consumption and labor cost. The findings of the present study demonstrated 50% lower daily dose, and 50% less nursing times. Similar results were reported for piperacillin CI in earlier studies.^[25]^ Duszynska et al^[26]^ observed a reduction of the total daily dose by 38% in patients with pneumonia including daily TDM. In a prospective, open-label, controlled study, total cost per treatment was reduced by 24% in patients with pneumonia and piperacillin CI encompassing all costs directly related to antibiotic use (drug, preparation, as well as treatment of adverse events).^[25]^ Considering that antibiotics are frequently affected by shortages less drug might help with emerging higher demands of intensive care treatment including antibiotic administration due to the corona crisis. Piperacillin-tazobactam, ampicillin-sulbactam, meropenem, cefotaxime, and cefepime are the most commonly reported antimicrobials in short supply in the United States which resembles the European situation.^[43–45]^ Additionally to the significant economic ramification, shortages affect patient care and outcomes by the use of broader-spectrum, more costly, less effective second-line, or more toxic agents.^[43,46]^ Furthermore, possible savings in nursing time exacerbated by protective measures due to COVID-19 to administer 1 or 2 doses (instead of 3 to 4 doses) when using CI, might also translate into relevant reduction of material cost and delay potential shortages.

There are several limitations of this study. First, the study was a single-center study which may have hampered robust estimates of the extent of PK variability. Second, CrCL was estimated using the Cockroft-Gault equation because CrCL measurement is not routinely performed in routine clinical care and ß-lactam clearance and Cockroft-Gault equation show a good overall correlation (*r* = 0.57).^[12]^ Third, this was a retrospective analysis of serum concentrations measured as total drug concentrations. Therapeutic cefotaxime exposure was calculated with an unbound faction of 50% considering the high protein binding. Finally, the study was relatively small, because the outbreak was stopped in the district of Heidenheim, Germany by May 2020.

## 5 Conclusions

Treatment of infections in critically ill patients remains a significant challenge for clinicians due to severely altered PK. The COVID-19 disease did not develop a significant influence on pharmacokinetic changes in critically ill patients with bacterial pneumonia. Our data strongly support a personalized dosing approach of ß-lactam antibiotics according to current recommendations including dosing software, CI, and TDM to improve therapeutic exposure. This personalized approach leads to high PK-target attainment within the first 48 hours of treatment as well as throughout the treatment course, while avoiding critically low ß-lactam concentrations. However, less drug, and reduction of nursing time with this approach might be beneficial in cases of COVID-19 to overcome emerging drug shortages and increased workload.

## Acknowledgments

The authors thank Dr. Anastasia Lemekhova for critically reviewing the manuscript.

## Author contributions

**Conceptualization:** Ute Chiriac, Otto R. Frey, Alexander Brinkmann.

**Data curation:** Otto R Frey, Anka C. Roehr, Andreas Koeberer, Patrick Gronau, Thomas Fuchs, Alexander Brinkmann.

**Formal analysis:** Ute Chiriac, Otto R. Frey.

**Investigation:** Ute Chiriac, Otto R. Frey.

**Methodology:** Ute Chiriac, Otto R. Frey, Alexander Brinkmann.

**Project administration:** Ute Chiriac, Otto R. Frey, Alexander Brinkmann.

**Software:** Ute Chiriac.

**Supervision:** Ute Chiriac, Otto R. Frey, Jason Roberts, Alexander Brinkmann.

**Validation:** Ute Chiriac, Otto R. Frey, Alexander Brinkmann.

**Visualization:** Ute Chiriac.

**Writing – original draft:** Ute Chiriac.

**Writing – review & editing:** Otto R. Frey, Anka C Roehr, Andreas Koeberer, Patrick Gronau, Thomas Fuchs, Jason Roberts, Alexander Brinkmann.
